# Non-Articular Osseous Sarcoidosis: A Rare Case of Active Sarcoidosis with Progressive Lung Lesions and Normal Inflammation Biomarkers

**DOI:** 10.3390/diagnostics15091135

**Published:** 2025-04-29

**Authors:** Jing Zhang, Yu Hu, Peixin Dong, Hefang Guo, Lixia Huang, Lili Chen, Yanbin Zhou

**Affiliations:** 1 Division of Pulmonary and Critical Care Medicine, The First Affiliated Hospital of Sun Yat-Sen University, Guangzhou 510080, China; zhangj265@mail.sysu.edu.cn (J.Z.); huyu27@mail2.sysu.edu.cn (Y.H.); dongpx3@mail2.sysu.edu.cn (P.D.); huanglix3@mail.sysu.edu.cn (L.H.); 2 Institute of Respiratory Diseases of Sun Yat-Sen University, Guangzhou 510080, China; 3 Department of Infectious Diseases, Hexian Memorial Hospital of PanYu District, Guangzhou 511400, China; 15271200127@163.com; 4 Department of Pathology, The First Affiliated Hospital of Sun Yat-Sen University, Guangzhou 510080, China

**Keywords:** sarcoidosis, angiotensin-converting enzyme, CRP, osseous, tuberculosis

## Abstract

Sarcoidosis is a rare multisystem inflammatory disease characterized by non-necrotizing granulomas, typically affecting the lungs, lymph nodes, skin, and bones. Due to its extreme clinical heterogeneity, diagnosis remains challenging. Within the skeletal system, the thoracic spine, ankles, and knees are the most commonly involved joints. We report a rare case of non-articular osseous sarcoidosis with progressive pulmonary lesions and persistently normal inflammatory biomarkers (ACE, CRP, ESR, IL-2, and TNF-α) that required differentiation from metastatic bone tumors and tuberculosis. Prior to presentation at our hospital, the patient did not respond to six months of anti-tuberculosis treatment and one month of systemic glucocorticoid therapy in three other hospitals. Based on lung and bone biopsies, she was finally diagnosed as having active sarcoidosis in our hospital. Despite 3 months of prednisone, pulmonary consolidation and bone lesions persisted until methotrexate was added. This case highlights the preference of combined glucocorticoid and methotrexate therapy for sarcoidosis with atypical osseous involvement and normal biomarkers, underscoring the urgent need for novel diagnostic tools to mitigate misdiagnosis.

**Figure 1:**
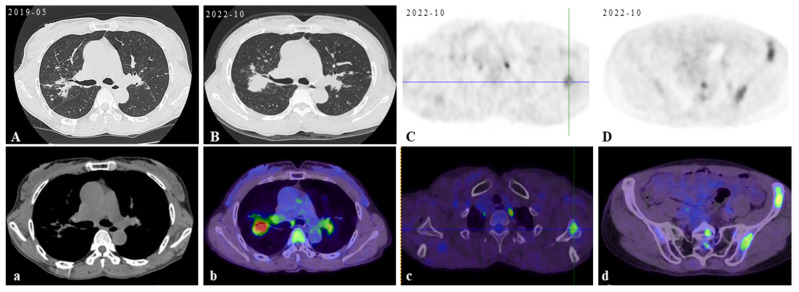
Initial scan: The chest CT scan on her first visit to our hospital displayed bilateral pulmonary nodules with calcified mediastinal/hilar lymphadenopathy (**A**,**a**). Three years later, her PET/CT images revealed progressive pulmonary consolidation and multiple hypermetabolic lymph nodes (**B**,**b**). Multiple hypermetabolic bone lesions were identified in the left scapula (**C**,**c**), left ischium (**D**,**d**), and so on. PET-CT scan colors represent high metabolic activity. A 46-year-old previously healthy female patient presented with cough and hemoptysis persisting for 2 years. In these 2 years, she had been treated in three hospitals sequentially. After six months of diagnostic anti-mycobacterium tuberculosis chemotherapy and one month of systemic glucocorticoid therapy, her follow-up chest CT scans showed that the lesions were not controlled. Therefore, she came to our hospital’s outpatient clinic for a consultation in May 2019. The chest CT in our hospital displayed bilateral pulmonary nodules with calcified mediastinal/hilar lymphadenopathy (**A**,**a**). However, the patient refused to be hospitalized for further examination and treatment. Over the following three years, her cough gradually worsened. In October 2022, her PET/CT displayed progressive consolidation in the lungs with multiple hypermetabolic lymph nodes. Multiple hypermetabolic foci were identified in the left scapula, left portions of the ribs, vertebral bodies, and left ischium (**B**–**D**,**b**–**d**). Therefore, she agreed to be admitted to our hospital in October 2022. There were no obvious abnormalities in blood routine, liver and kidney function, CRP (C-reactive protein), ESR (erythrocyte sedimentation rate), ACE (angiotensin-converting enzyme), IL-2, TNF-α, LDH (lactate dehydrogenase), ALP (alkaline phosphatase), tumor markers, serum cryptococcal capsular antigen, aspergillus galactomannan antigen, 1,3-β-D-glucan, the PPD-test, antinuclear antibody spectrum, antineutrophil cytoplasmic antibody, electrocardiogram, echocardiography, and nasopharynx magnetic resonance imaging (MRI). The pelvis MRI identified irregular patchy abnormal signals in the ilium (Figure 2A), left ischium (Figure 2B), and sacrum (Figure 2C). A bronchoscopy was performed with lavage in the apical segment of the right upper lobe and biopsy in the lingular segment of the left upper lobe. The microbiological (bacterial, fungal, and TB) cultures, acid-fast bacilli smear, quantitative detection of TB DNA, fungal GM antigen test, and metagenomic next-generation sequencing (mNGS) were all negative. The bronchoscopy lung biopsy revealed non-caseating granulomas (Figure 3A), which was in accordance with sarcoidosis, with negative results of the acid-fast staining (Figure 3B), microbiological cultures, quantitative detection of TB DNA, and mNGS. According to previous reports, active sarcoidosis is usually characterized by the elevation of inflammation biomarkers including ACE, CRP, ESR, IL-2, and TNF-α. Among these biomarkers, the level of ACE, which has been proven to be associated with the burden of granulomas and disease activity, establishes the auxiliary diagnostic criterion for sarcoidosis [1,2]. Meanwhile, all the inflammatory markers were normal in this patient, and she did not respond to one month of systemic glucocorticoids in previous hospitals. In addition, it is well-known that osseous sarcoidosis predominantly affect the joints, such as the thoracic spine, ankles, and knees [3], whereas our patient’s manifestations were mainly focused on non-articular bone such as the scapula, ribs, and ischium, which mimicked metastatic advanced lung cancer. Recent studies have demonstrated that patients with sarcoidosis exhibit an elevated risk of developing various malignancies, including hematologic and solid malignancies [4]. This association is thought to arise from chronic inflammation, immune dysregulation, shared etiologic factors, and genetic predisposition [5]. Notably, the correlation between sarcoidosis and lymphoma is particularly significant, observed both preceding and following the diagnosis of sarcoidosis. This may be mediated by lymphoproliferative processes linked to heightened mitotic activity in chronic active disease, culminating in the rare sarcoidosis–lymphoma syndrome [4]. Furthermore, sarcoidosis-like granulomas have been documented in 14% of Hodgkin’s lymphoma cases and 7% of non-Hodgkin’s lymphoma cases [6,7], underscoring the diagnostic complexity of granulomatous findings. Given these diagnostic challenges, the presence of non-caseating granulomas in a single biopsy cannot definitively confirm sarcoidosis. To address this uncertainty, additional left iliac bone and thoracoscopic lung biopsies were performed. These biopsies revealed no evidence of tuberculosis, fungal infection, or malignancies, with negative results for TB DNA, mNGS, acid-fast staining (Figure 3C), and methenamine silver staining (Figure 3D). This indicates that new biomarkers or novel diagnostic tools need to be explored to reduce clinical misdiagnosis in the future. Previous studies showed that the JAK/STAT signaling pathway and mTOR signaling pathway, which play a crucial role in granuloma formation, were promising biomarkers in predicting diagnosis and treatment response of sarcoidosis [8]. However, neither has yet achieved the ideal sensitivity and specificity for widespread clinical adoption [9]. In recent years, radiomic artificial intelligence (AI) technologies, typified by deep learning, have been developed to improve the accuracy and precision of sarcoidosis diagnosis, providing new insights into the mechanisms of sarcoidosis [10]. With the development of computers’ computing power, AI technologies will probably replace ACE as the new diagnostic criterion for sarcoidosis in the future. In our patient, considering that there were no life-threatening or organ-threatening situations and an unacceptably impaired quality of life, we decided to restart and extend the duration of glucocorticoid (0.5 mg/kg/d prednisone, 35 mg/day) from November 2022 (Figure 4A,a). However, pulmonary consolidation and bone lesions were not absorbed after three months of treatment with prednisone (Figure 4B,b). Therefore, 15 mg QW [3] methotrexate was added to improve the therapeutic efficacy. After the prescribed combination of prednisone and methotrexate was administered for three months, pulmonary consolidation was noticeably absorbed (Figure 4C,c). Due to presenting a full-moon face, the patient requested a reduction in the steroid dosage. Then, the reduction in the prednisone dose to 20 mg/day triggered relapse (Figure 4D,d), reversed by reinstating the prednisone dose to 35 mg/day with methotrexate (Figure 4E,e). This indicated that the combination of prednisone and methotrexate should be taken as the preferred therapy for sarcoidosis patients with normal inflammatory markers and non-articular osseous sarcoidosis. Unfortunately, the patient’s PETCT could not be re-examined to show the absorption of osseous sarcoidosis because the patient died in a traffic accident in December 2024.

**Figure 2:**
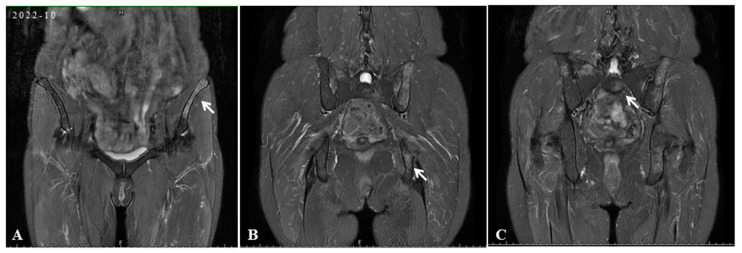
In the initial scan, pelvis MRI identified irregular patchy abnormal signals in the ilium (white arrow) (**A**), left ischium (white arrow) (**B**), and sacrum (white arrow) (**C**).

**Figure 3:**
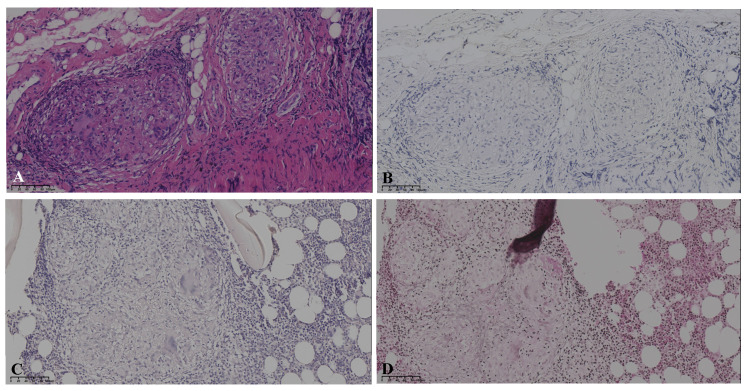
Pathology: The bronchoscopy lung biopsy revealed non-caseating granulomas in hematoxylin-eosin staining (**A**), with negative results of acid-fast staining (**B**). The left iliac bone biopsy showed the formation of granulomas with negative results for acid-fast staining (**C**) and methenamine silver staining (**D**).

**Figure 4:**
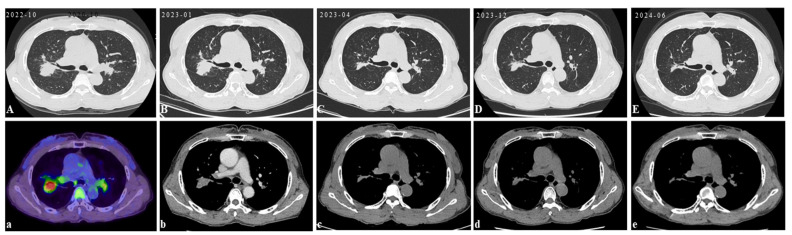
Follow-up: The above CT images were taken from October 2022 to June 2024 during treatment. The PETC/CT before treatment displayed progressive pulmonary consolidation and multiple hypermetabolic lymph nodes. PET-CT scan colors represent high metabolic activity (**A**,**a**). Following 3 months of prednisone monotherapy (35 mg/day), pulmonary consolidation was not noticeably absorbed (**B**,**b**). Subsequent adjunctive methotrexate (15 mg QW) led to the marked absorption of lung lesions within 3 months (**C**,**c**). The reduction in the prednisone dose to 20 mg/day triggered relapse (**D**,**d**), reversed by reinstating the prednisone dose to 35 mg/day with methotrexate (**E**,**e**).

## Data Availability

Further inquiries should be directed to the corresponding author.

## References

[1] Vorselaars A.D., van Moorsel C.H., Zanen P., Ruven H.J., Claessen A.M., van Velzen-Blad H., Grutters J.C. (2015). ACE and sIL-2R correlate with lung function improvement in sarcoidosis during methotrexate therapy. Respir. Med..

[2] Gupta R., Judson M.A., Baughman R.P. (2022). Management of Advanced Pulmonary Sarcoidosis. Am. J. Respir. Crit. Care Med..

[3] Smedslund G., Kotar A.M., Uhlig T. (2022). Sarcoidosis with musculoskeletal manifestations: Systematic review of non-pharmacological and pharmacological treatments. Rheumatol. Int..

[4] Patt Y.S., Ben-Shabat N., Sharif K., Patt C., Elizur Y., Arow M., Cohen A.D., Watad A., McGonagle D., Amital H. (2024). The Association Between Sarcoidosis and Malignancy: A Comprehensive Population-Based Cohort Study. J. Clin. Med..

[5] Brito-Zerón P., Flores-Chávez A., González-de-Paz L., Feijoo-Massó C., de Escalante B., González-García A., Gómez-de-la-Torre R., Policarpo-Torres G., Alguacil A., García-Morillo J.S. (2024). Temporal relationship between sarcoidosis malignancies in a nationwide cohort of 1942 patients. Postgrad. Med. J..

[6] Alzghoul B.N., Zayed Y., Obeidat A., Alzghoul B., Naser A., Shilbayeh A.R., Innabi A., Al-Hakim T., Buchanan M., Mehrad B. (2021). Clinical Characteristics of Sarcoidosis Patients with Self-Reported Lymphoma: A US Nationwide Registry Study. Lung.

[7] Matsuo T., Tanaka T., Omote R., Okada T., Notohara K., Okada K. (2022). Diffuse large B-cell lymphoma in the course of systemic sarcoidosis: A case report and review of 30 Japanese patients with sarcoidosis-lymphoma syndrome. J. Clin. Exp. Hematop..

[8] Bennett D., Bargagli E., Refini R.M., Rottoli P. (2019). New concepts in the pathogenesis of sarcoidosis. Expert. Rev. Respir. Med..

[9] Silva M., Nunes H., Valeyre D., Sverzellati N. (2015). Imaging of Sarcoidosis. Clin. Rev. Allergy Immunol..

[10] Azoulay L.D., Mei X., Fauveau V., Liu Z., Robson P., Levi S., Devesa A., Trivieri M.G., Fayad Z. (2025). Deep learning approaches to predict late gadolinium enhancement and clinical outcomes in suspected cardiac sarcoidosis. Sarcoidosis Vasc. Diffuse Lung Dis..

